# Preschool Phonological and Morphological Awareness As Longitudinal Predictors of Early Reading and Spelling Development in Greek

**DOI:** 10.3389/fpsyg.2017.02039

**Published:** 2017-11-27

**Authors:** Vassiliki Diamanti, Angeliki Mouzaki, Asimina Ralli, Faye Antoniou, Sofia Papaioannou, Athanassios Protopapas

**Affiliations:** ^1^Department of Primary Education, University of Crete, Rethymno, Greece; ^2^Department of Special Needs Education, University of Oslo, Oslo, Norway; ^3^Department of Philosophy, Pedagogy, and Psychology, National and Kapodistrian University of Athens, Athens, Greece; ^4^Department of Medicine, University of Crete, Rethymno, Greece

**Keywords:** morphological awareness, phonological awareness, reading, spelling, longitudinal study

## Abstract

Different language skills are considered fundamental for successful reading and spelling acquisition. Extensive evidence has highlighted the central role of phonological awareness in early literacy experiences. However, many orthographic systems also require the contribution of morphological awareness. The goal of this study was to examine the morphological and phonological awareness skills of preschool children as longitudinal predictors of reading and spelling ability by the end of first grade, controlling for the effects of receptive and expressive vocabulary skills. At Time 1 preschool children from kindergartens in the Greek regions of Attika, Crete, Macedonia, and Thessaly were assessed on tasks tapping receptive and expressive vocabulary, phonological awareness (syllable and phoneme), and morphological awareness (inflectional and derivational). Tasks were administered through an Android application for mobile devices (tablets) featuring automatic application of ceiling rules. At Time 2 one year later the same children attending first grade were assessed on measures of word and pseudoword reading, text reading fluency, text reading comprehension, and spelling. Complete data from 104 children are available. Hierarchical linear regression and commonality analyses were conducted for each outcome variable. Reading accuracy for both words and pseudowords was predicted not only by phonological awareness, as expected, but also by morphological awareness, suggesting that understanding the functional role of word parts supports the developing phonology–orthography mappings. However, only phonological awareness predicted text reading fluency at this age. Longitudinal prediction of reading comprehension by both receptive vocabulary and morphological awareness was already evident at this age, as expected. Finally, spelling was predicted by preschool phonological awareness, as expected, as well as by morphological awareness, the contribution of which is expected to increase due to the spelling demands of Greek inflectional and derivational suffixes introduced at later grades.

## Introduction

Reading and spelling are considerable cognitive undertakings that require the integration of written and spoken language. An overwhelming body of research evidence suggests that children’s phonological awareness, which requires conscious reflection upon and explicit manipulation of the constituent speech sounds of language, is a necessary requirement for the acquisition of the alphabetic principle (Byrne, 1996) and a key skill for mastering decoding (National Reading Panel, 2000; Lonigan et al., 2009) and spelling across orthographies (Cataldo and Ellis, 1988; Ellis and Cataldo, 1990; Byrne and Fielding-Barnsley, 1991, 1993; Porpodas, 1999; Aidinis and Nunes, 2001; Caravolas et al., 2001, 2005; Cardoso-Martins and Pennington, 2004; Furnes and Samuelsson, 2010).

On the other hand, morphological awareness plays a fundamental role in mastering decoding, reading fluency, and comprehension (Deacon and Kirby, 2004; Kuo and Anderson, 2006; Tong et al., 2011; Kirby et al., 2012; Deacon et al., 2014; Muroya et al., 2017) and orthographic spelling (Deacon and Kirby, 2004; Deacon and Bryant, 2005, 2006; Desrochers et al., 2017) across orthographies (Wei et al., 2014; Rothou and Padeliadu, 2015; Grigorakis and Manolitsis, 2016; Pan et al., 2016; Vaknin-Nusbaum et al., 2016a,b; Muroya et al., 2017). Morphological awareness refers to (a) an explicit understanding of morphological relations between word forms and meanings, such as grammatical inflection and productive derivation, and (b) the ability to manipulate the morphological structure of words (Carlisle, 1995). The present study aimed to examine the predictive value of preschool morphological and phonological awareness in learning to read and spell.

### Morphological Awareness and Literacy Development

It has been forcefully argued that reading comprehension cannot succeed unless the reader appreciates morphological word formation, that is, how differences in word forms relate to differences in meaning (Carlisle, 2003). This suggests that an explicit understanding of morphological relations, termed morphological awareness, is a prerequisite to skilled reading. In fact morphological awareness is related not only to reading comprehension, but also to spelling (e.g., Deacon et al., 2009; Casalis et al., 2011), vocabulary (McBride-Chang et al., 2005; Sparks and Deacon, 2015), and word and pseudoword reading (Deacon and Kirby, 2004; Kirby et al., 2012). The contribution of morphological awareness to spelling is robust to a multitude of control variables (Deacon et al., 2009) and includes both inflected and derived forms (Deacon et al., 2010) beyond the spelling of specific morphemes (Casalis et al., 2011).

Deacon and Kirby (2004) examined the role of both phonological and morphological awareness in learning to read for English-speaking Canadian children. They investigated the longitudinal prediction of Grades 3, 4, and 5 pseudoword reading, single word reading, and reading comprehension from Grade 2 phonological and morphological awareness. They found that morphological awareness made a small but unique contribution to all aspects of reading development – mainly pseudoword reading and reading comprehension – during the 3 years of middle elementary school, over and beyond the effect of phonological awareness. They argued that morphological awareness might have accounted for more variance in the reading variables if multiple measures of various formats and tapping a broader range of derivations and inflections had been used. The present study addressed this methodological limitation by assessing children in an elaborate and systematic battery of phonological and morphological awareness tasks.

In another study of English-speaking Canadian children, Deacon et al. (2009) examined the predictive value of morphological awareness, assessed in the early school years, for the prediction of spelling, assessed in middle elementary grades. They reported that Grade 2 morphological awareness accounted for approximately 8% of the variance in Grade 4 general spelling skills, beyond the effect of verbal and non-verbal intelligence, phonological awareness, verbal short-term memory, and rapid-automatized naming (RAN).

Few studies have studied the contribution of morphological awareness assessed before the onset of formal reading instruction. Casalis and Louis-Alexandre (2000) studied the longitudinal contribution of phonological and morphological awareness to decoding and reading comprehension. They assessed French-speaking kindergarten children in a variety of morphological awareness tasks measuring both inflectional and derivational morphology. Their findings showed strong correlations between phonological and morphological awareness tasks, as well as unique contributions of both skills to Grade 2 decoding skills and reading comprehension. However, they only analyzed the correlations for individual tasks and did not examine the overall effects of morphological and phonological awareness skills by considering all the corresponding tasks together. Therefore, the total magnitude of the longitudinal relationship remained unknown.

More recently, using latent variable modeling in Chinese, Pan et al. (2016) found that pre-literate syllable and morphological awareness predicted character reading, reading fluency, reading comprehension, and writing at the age of 11 years, beyond any effects of phonological awareness, but only indirectly, that is, through post-literate morphological awareness assessed at the ages of 7–10 years.

The longitudinal relation between early morphological awareness and reading and spelling skills has also been studied in Greek. Manolitsis (2006) found that morphological awareness, assessed in kindergarten, longitudinally predicted Grade 1 word reading but its contribution to accuracy was not significant when kindergarten phonological awareness was controlled for. Pittas and Nunes (2014) assessed first and third graders in three morphological awareness tasks: a pseudoword inflection task, a sentence analogy task, and a morphological relatedness task. They found a unique contribution of morphological awareness to reading – but not to spelling – assessed 8 months later, even after partialling out the effects of grade, verbal ability, phonological awareness, and initial reading level.

Grigorakis and Manolitsis (2016) examined the longitudinal prediction of Greek morphological spelling from morphological awareness measured before and at the beginning of formal literacy instruction. They assessed 229 kindergarten children 5–6 years old on a variety of morphological awareness tasks measuring their ability to recognize and manipulate inflections, derivations, and compound words. Spelling of inflectional suffixes in words and pseudowords was assessed at Grades 1 and 2. Morphological awareness was a significant longitudinal predictor of word spelling, surviving control for verbal and non-verbal intelligence, verbal short-term memory, receptive and expressive vocabulary, letter sound knowledge, RAN, and phonological awareness.

Finally, in a cross-linguistic study comparing English, French, and Greek, Desrochers et al. (2017) found that Greek children’s morphological awareness skills at the beginning of Grade 2 were unique predictors of reading comprehension and spelling, but not of reading accuracy – as in English – and fluency – as in both English and French – at the end of the same grade.

Evidence for the importance of morphological awareness has also been provided by intervention studies. If morphological awareness forms a critical substrate for reading development, then training in morphological awareness, if successful, should lead to measurable improvements in reading performance. Due to their experimental – rather than correlational – nature, studies of morphological awareness training constitute an empirically crucial source of evidence regarding the connection between morphological awareness and literacy. Indeed, instruction in morphological awareness has been shown to result in benefits across literacy domains, especially when combined with phonological awareness training (e.g., Lyster, 2002; Lyster et al., 2016; Manolitsis, 2017; see meta-analyses and systematic reviews in Reed, 2008; Bowers et al., 2010; Carlisle, 2010; Goodwin and Ahn, 2010, 2013).

However, even though dozens of morphological awareness studies have accumulated to date, as seen in the aforementioned reviews, a confident conclusion remains unwarranted because it has been challenging to establish the specificity of training. The majority of studies have failed to employ an active control group receiving instruction of similar structure and intensity but non-morphological in content. Indeed, many studies have simply compared the experimental group to a passive control group not receiving any special instruction but following the regular classroom program. When active control groups are employed the benefits to literacy from morphological training are not significantly stronger (e.g., comparing against phonological awareness training; Lyster, 2002; Lyster et al., 2016).

An additional difficulty with the theoretical interpretation of the majority of these training studies is that they have relied, at least in part, on printed materials or strategies potentially exploiting the orthographic knowledge of participants, thereby obscuring the origin of the observed effects. That is, although the focus of the instruction was on the morphological aspects of words, if training took place using written words then children may have exhibited literacy gains due to the fact that they received a form of reading or spelling instruction rather than to morphological awareness *per se*.

In sum, despite the recent surge in interest in the relationship between morphological awareness and reading skill development, and the strong evidence for its importance, the relevant literature has not conclusively established the precedence, or necessity, of morphological awareness for reading development and for particular reading skills. Many studies have examined concurrent correlations and most have assessed children in elementary grades, for which reciprocal effects may have contributed to the reported findings. That is, if morphological awareness is assessed after the onset of reading instruction, it is possible that exposure to the various printed word types may have contributed to the further development of morphological awareness. Therefore, a finding of robust correlations may conceivably be due to an inverse direction of causation than typically hypothesized.

Although longitudinal studies are one step toward addressing this shortcoming, it is also critical that the first assessment of morphological awareness takes place before the onset of reading instruction, to minimize effects of exposure to print. This requires the development and validation of appropriate testing materials for preschoolers that arguably address metalinguistic morphological skills. In the present study we have thus examined the longitudinal prediction of early (Grade 1) reading skills by preschool morphological awareness, controlling for phonological awareness and vocabulary. To obtain a more nearly complete picture of the importance of morphological awareness for reading skill development, we have applied a comprehensive battery of reading outcomes, including word and pseudoword accuracy, reading fluency, reading comprehension, as well as spelling.

### Development and Assessment of Morphological Awareness

Typical language development involves unconscious use of morphology. Very young children produce overgeneralizations, such as “buyed” (instead of “bought”). The production of these errors suggests a gradual development in understanding the rules of inflectional morphology (Berko, 1958; Selby, 1972). Nonetheless, the boundary between tacit knowledge of morphological processes and conscious morphological awareness has not been sufficiently investigated. In many cases it is not clear whether differences in measures of morphological awareness reflect differences in metalinguistic awareness or in implicit morphological knowledge (Nagy et al., 2014). Metalinguistic awareness is thought to be a special kind of linguistic functioning, beyond language acquisition, which develops in middle childhood (Tunmer et al., 1984).

The morphological processes of grammatical inflection and productive derivation seem to follow a similar but non-simultaneous developmental progression. Evidence shows that awareness of inflectional morphology is acquired in the first school years (Diakogiorgi et al., 2005; Kuo and Anderson, 2006), whereas awareness of derivational morphology develops toward the fourth year (Anglin, 1993; Carlisle, 2000), and continues to grow throughout the school years (Berko, 1958; Anglin, 1993; Berninger et al., 2010). Carlisle (1995) suggested that children’s awareness of derivational morphology makes a transition from an implicit to an explicit level at the ages of kindergarten and first grade.

Morphological awareness tasks have been classified according to their cognitive and meta-cognitive requirements, which may operate at either an implicit or an explicit level (Deacon et al., 2008). Lexical judgment tasks, which require children to decide whether two words are related or not, have been widely used to assess implicit morphological skills (e.g., Mahony et al., 2000; Duncan et al., 2009), whereas analogy and production tasks have been used to tap explicit skills (e.g., Berko, 1958; Derwing, 1976; Nunes et al., 1997; Carlisle, 2000; Kirby et al., 2012). Production tasks have also been differentiated between implicit and explicit (Casalis and Louis-Alexandre, 2000).

Diamanti et al. (in press) recently examined the development of morphological awareness in Greek children 4–7 years old. They compared the domains of inflectional and derivational morphology, adopting a distinction between two levels, namely epilinguistic control and metalinguistic awareness. Epilinguistic control refers to an intermediate level of elementary awareness that has been posited to intervene developmentally between the acquisition of the linguistic skill and the acquisition of metalinguistic awareness (Gombert, 1992). In contrast, metalinguistic awareness refers to the individual’s ability to reflect upon and consciously manipulate morphemes, as well as the ability to deliberately apply word formation rules. Following Carlisle (1995), epilinguistic control is evidenced in judgment tasks, whereas full-blown metalinguistic awareness is evidenced in production tasks (see Diamanti et al., in press, for further discussion). In addition to the expected performance increase with age, Diamanti et al. (in press) found that a single factor sufficed and accounted for 0.59 of the variance in the four tasks, consistent with a common developmental path underlying both domains and both levels of morphological awareness. In comparison of the developmental growth curves among tasks, they found that production of derivational morphemes was more difficult than production of inflectional morphemes and judgment of derivational morphemes, whereas the differences between the two inflectional tasks and between the two judgment tasks were not significant.

Given these findings, Diamanti et al. (in press) suggested that at these ages epilinguistic control is similarly effective for the two morphological domains whereas full metalinguistic awareness of derivational morphology trails behind that of inflectional morphology, at least as measured by these specific tasks. Thus, on the one hand, this study highlighted the need for early tracking and distinctions among levels and domains of morphological awareness. On the other hand, it demonstrated the reliability and validity of the materials used and the potential of this combination of subscales to form a reliable and coherent scale for overall wide-range assessment of morphological awareness in the preschool and early elementary school age range. The present study is a follow-up of a subset of the children in that study, who attended preschool at the time and were assessed again 1 year later, in Grade 1, on reading-related outcome variables.

### Relevant Properties of Greek

This subsection is reproduced from Diamanti et al. (in press). Greek is a language with rich inflectional and derivational morphology (see Ralli, 2003) and relatively consistent orthography (Protopapas and Vlahou, 2009). Nouns and adjectives are obligatorily inflected for gender, number, and case via fusional suffixation. For example, the noun χoρóς (/xoros/ “dance”) is composed of the stem χoρ – (/xor/ expressing the core semantics) and the inflectional suffix – oς (/os/ signifying masculine singular nominative case). Verb forms also include a stem and an obligatory inflectional ending, both of which may be simple or complex. Verbs are inflected for voice, aspect, tense, number, and person [Ralli, 2003; see Klairis and Babiniotis (2004) and Holton et al. (2012) for comprehensive descriptions]. For example, the verb χoρ𝜀ύω (/xorevo/ “I dance”) is composed of the same stem χoρ – (/xor/), the derivational affix – 𝜀ύ – (/ev/ forming a verb from a noun), and the inflectional suffix – ω (/o/ signifying first person singular).

Distinct inflectional classes are recognized for both nouns/adjectives and verbs, each with its own set of suffixation and stem alternation rules (Ralli, 2003, 2005; Holton et al., 2012). Word formation in Greek also includes systematic derivational processes, especially for nouns (based on verb stems) and adjectives (based on verb and noun stems). Compounding is also highly productive, as new adjectives, nouns, and verbs can be created from existing stems and words [see Ralli (2003, 2005) for more information].

Morphology has extensive orthographic consequences in Greek, insofar as derivational and grammatical suffixes are associated with specific spellings, which also serve to disambiguate homonyms. Knowledge of the inflectional type is often required for correct spelling of adjective, noun, and verb suffixes [see Protopapas (2017) for more information and references]. Therefore, it seems reasonable to hypothesize that an understanding of morphological processes will be especially beneficial in learning to spell, and particularly useful in spelling the inflectional suffixes (Grigorakis and Manolitsis, 2016). This is important in light of the fact that Greek morphological spelling is known to be challenging, including both inflectional and – especially – derivational suffixes (Protopapas et al., 2013a; Diamanti et al., 2014).

A small amount of instructional activity related to morphological awareness takes place informally in the Kindergarten curriculum as part of vocabulary instruction, in the context of shared book reading and retelling, including discussion about word types such as diminutive derivation and number inflection, along with phonological awareness activities such as letter–sound association and identification. Systematic decoding is taught in Grade 1, so that most children are able to read by mid-grade, after which point some instruction related to morphological awareness appears, for example teaching the distinct spellings of noun and verb vowel endings (i.e., inflectional suffixes).

Most Greek children have mastered the inflectional paradigms of the language to a large extent by the age of entering elementary education, at least as far as the suffixes with orthographic consequences are concerned (i.e., case, gender, and number, for adjectives and nouns, and person and number, for verbs). Normally developing kindergarten children approach ceiling performance in the production of verb past tense and noun gender, number, and case (Mastropavlou, 2006) although persistent difficulties with verb aspectual formation and noun gender are observed in certain word classes with unusual properties (Stavrakaki and Clahsen, 2009; Varlokosta and Nerantzini, 2013, 2015). Thus, morphological acquisition is largely but not entirely completed by Grade 1.

## Materials and Methods

### Participants

The study sample consisted of 104 children (54 girls and 50 boys) assessed at the middle of kindergarten (February–March; age *M* = 67.3 months; *SD* = 3.6) and again at the end of Grade 1 (April–May; about 14 months later). They were native speakers of Greek and did not have any diagnosed developmental delay or emotional disorder prohibiting them from enrollment in typical (general) education settings. They were recruited from schools in rural (17%), semi-urban (19%), and urban (63%) areas of four geographically dispersed provinces of Greece, including a variety of socioeconomic and ethnic backgrounds. Sample demographics represent a close approximation to the Greek population (77% urban and 23% rural) based on the 2011 census.

Permission to conduct the study in these public schools was granted by the Ministry of Education following formal review and approval of the study plan by the Research Office of the Educational Policy Institute. Parental and school approval, as well as the child’s oral assent, were obtained prior to test administration. Participants were not specifically selected; rather, consent forms were distributed to entire classrooms and children who returned the signed parental consent were included in the study.

### Materials

Time 1 (predictor) measures included receptive and expressive vocabulary, and phonological and morphological awareness. These tasks were administered through an Android application (app) for mobile devices (tablets) featuring automatic application of ceiling rules. Time 2 (outcome) measures included word and pseudoword reading accuracy, text reading fluency and comprehension, and spelling. The four reading outcome measures were from “ΔAΔA,” a standardized reading test by Padeliadu et al. (in press).

#### Receptive Vocabulary

Four different images were displayed while a recorded spoken word was played out by the app, and the child was asked to choose the image that best represented the word that was heard. The three other images corresponded to a word from the same semantic category, a phonologically similar word, and an unrelated word. Words were appropriate for children in preschool and early elementary grades (including animals, objects, actions, adjectives, abstract concepts, etc.) and were presented in order of increasing difficulty (determined by Rasch analysis of pilot data from 237 children on 65 original items). Scoring was recorded automatically, amounting to the number of correct responses. The number of items was *N* = 30 and the reliability of the scale (Cronbach’s coefficient of internal consistency) was α = 0.88.

#### Expressive Vocabulary

This was a word definition task, in which each child was asked to give a brief definition of a series of words. Words were selected to cover a range of abilities for children in preschool and early elementary grades, including a variety of semantic and grammatical categories (animals, food, professions, objects, actions, abstract concepts, etc.), based on the results of a pilot study (parallel to that for receptive vocabulary, with 50 original items). Manual off-line scoring matched other similar tasks (i.e., WISC vocabulary), such that a proper word definition received two points, whereas examples of word use or descriptions were scored with 1 or 0, depending on word understanding and richness of expression (*N* = 28; α = 0.91).

#### Phonological Awareness

This was a composite score corresponding to the total number of items correctly responded to in a series of eight tasks assessing initial syllable matching (*n* = 7 items; Cronbach’s α = 0.84), initial phoneme matching (*n* = 7; α = 0.84), syllable blending (*n* = 5; α = 0.89), phoneme blending (*n* = 7; α = 0.93), syllable segmentation (*n* = 6; α = 0.95), phoneme segmentation (*n* = 7; α = 0.95), syllable deletion (*n* = 7; α = 0.94), and phoneme deletion (*n* = 7; α = 0.92). For the total scale, as entered in the analyses, *N* = 53, α = 0.97.

In the initial syllable (or phoneme) matching tasks, children heard the label of a displayed target image and the labels of three other simultaneously displayed images and had to choose which of the three images began with the same syllable (phoneme) as the target image. In the blending tasks, children had to compose words from a series of syllables (phonemes) that were heard individually. In the syllable (phoneme) segmentation tasks children heard a word and were then asked to pronounce the individual syllables (phonemes) it comprised. Finally, in the syllable (phoneme) deletion, children were asked to listen carefully to a word and then to repeat it omitting a specific syllable (phoneme).

#### Morphological Awareness

This was a composite score corresponding to the total number of items correctly responded to in a series of three tasks assessing judgment (*n* = 8 items; α = 0.80) and production (*n* = 11 items; α = 0.73) of inflectional suffixes and production of derivational suffixes (*n* = 16 items; α = 0.94). For the total scale, as entered in the analyses, *N* = 35, α = 0.93. The following description of the tasks is based on Diamanti et al. (in press).

##### Inflectional morphemes judgment task

Children saw a picture displaying either one or two turtles performing an action while listening to two sentences spoken by two penguin figures displayed next to the action picture. Children had to choose the sentence matching the picture by pointing at one of the two penguins after hearing the sentences. Each pair of sentences contained one pseudoword differing in inflectional suffix, which was either singular or plural. For example, given a picture of two turtles taking photographs, the two sentences were “the turtles *skeni*_3rd.sg_ photos” and “the turtles *skenoun*_3rd.pl_ photos.” The correct sentence is the second one because the inflectional suffix of the pseudoverb denotes the plural form and agrees with the subject, thus matching the picture. Given a picture of a turtle holding two rulers, the two sentences were “the turtle is holding the_acc.sg_ *serapa*_acc.sg_” and “the turtle is holding the_acc.pl_ *serapes*_acc.pl_” (the critical pseudoword is denoted by italics). The correct sentence is the second one because the inflectional suffix of the pseudonoun denotes the plural form and matches the picture.

##### Inflectional morphemes production task

Children saw a pair of pictures, illustrating actions performed by turtles differing in the number of agents or patients of the depicted action, while listening to a verbal description including a pseudoword (a pseudoverb in eight sentences, for the action, and a pseudo-noun in three sentences, for the object). Children were then provided with the beginning of a second sentence, matching the second picture, up to the subject of the verb, and were asked to change the pseudoword number (from singular to plural or from plural to singular) accordingly. For example, given a picture of two turtles with sunglasses and a picture of one turtle with sunglasses, the sentence and prompt would be “The turtles *menane*_3rd.pl_ glasses. The turtle…” and the child should say “*menai*_3rd.sg_ glasses”; given a picture of a turtle waving at a monkey and a picture of a turtle waving at two monkeys, the sentence and prompt would be “The turtle is greeting the_acc.sg_ *reipou*_acc.sg_. The turtle is greeting the_acc.pl_” and the child should say “*reipoudes*_acc.pl_” (the critical pseudoword is denoted by italics).

##### Derivational morphemes production task

Children saw a picture while listening to a sentence with a critical word (a different one for each sentence) and the beginning of a second sentence that was syntactically altered and required manipulation of a derivational morpheme on the critical word to be completed correctly (e.g., “The sea deepens. The sea is…” requiring “deep”; “Miriam always teases her friends. Miriam is a…” requiring “teaser” /piraxtiri/, derived from /pirazo/). The task targeted a variety of derivational morphemes, denoting property, profession, establishment/institution, material, collection, comparatives, action, device, nationality/origin, etc.

#### Word Reading Accuracy

The word decoding test of the ΔAΔA decoding subscale was used, which consists of 57 words two to seven syllables long, with gradually increasing number of syllables and semantic complexity and decreasing frequency of occurrence, printed vertically. Words were nouns, adjectives, passive participles, and verbs. A stopping criterion of five consecutive errors was applied. The number of words read correctly was noted. The internal consistency of the entire “decoding” factor of ΔAΔA (which also includes pseudoword decoding, word/pseudoword discrimination, and word identification) as reported for elementary grades is high (ω = 0.90, *H* = 0.91).

#### Pseudoword Reading Accuracy

The pseudoword decoding subtest of the ΔAΔA decoding subscale was used, which consists of 40 non-words two to six syllables long, with gradually increasing number of syllables and phonological complexity, printed vertically. A stopping criterion of five consecutive errors was applied. The number of non-words read correctly was noted.

#### Reading Fluency

A grade-appropriate 247-word passage with an ancient Greek mythological theme from the reading fluency subscale of ΔAΔA was used. Children were asked to read the passage as quickly and as accurately as they could. The score of the test was the number of words read correctly within 1 min.

#### Reading Comprehension

The first three passages from the reading comprehension subscale of ΔAΔA were used, which were short and appropriate for the age of the participants, with gradually increasing semantic and syntactic difficulty. The first and second passages were narratives, while the third one was expository. Children had to answer seven multiple-choice questions for each passage while having the texts available. The questions required meaning abstraction based on vocabulary knowledge, as well as literal and inferencing skills. The score was the total number of questions answered correctly for all three passages (out of a total of 21 questions). The internal consistency of the entire “comprehension” factor of ΔAΔA (which includes three more passages, for a total of six) as reported for elementary grades is satisfactory (ω = 0.89, *H* = 0.64).

#### Spelling

Spelling ability was assessed using a standardized spelling-to-dictation test (Mouzaki et al., 2010), which includes 60 words dictated in isolation and in a sentence at a child-determined pace. A stopping criterion of six consecutive errors was applied. Each word was scored with one point for accurate spelling.

### Procedure

All measures were administered individually by specially trained research assistants, following a common procedure, in a quiet room at the children’s kindergarten (Time 1) or school (Time 2). Time 1 (predictor) measures were administered in two to three sessions of 40–45 min within 2 weeks (in the context of a variety of other tasks not reported here) using a tablet app custom made for this purpose. All visual and auditory stimuli were provided by the app as images and pre-recorded utterances. Scoring was automated when possible (i.e., evaluation of selection accuracy), or entered manually after administration when human judgment was necessary (i.e., evaluation of spoken responses). Time 2 (outcome) measures were administered individually in one 40–45-min-long session in the traditional (paper and pencil) format.

## Results

There were no missing data for this group of participants (*N* = 104 in all analyses). Visual examination of univariate quantile–quantile plots and bivariate scatterplots revealed six extreme outliers (two low values in receptive vocabulary, one low and one high in fluency, and two high in spelling), which were replaced by winsorized values at the appropriate percentile (1/*N* for single values and 2/*N* for two values) in order to retain a full data set. **Table 1** displays descriptive statistics following this minor cleanup. Despite some mild deviations from normality, no extreme values of skew or kurtosis were observed. **Table 2** displays the intercorrelations among all variables. Age was not significantly correlated with morphological awareness (*r* = 0.033, *p* = 0.740) or with any of the outcome variables (all *p* > 0.11), probably due to the restricted age range in this sample. Therefore, age was not entered as a predictor in the regression models.

**Table 1:** Descriptive statistics for predictor and dependent variables.

							Shapiro–Wilks			
Variable	*M*	*M*%	mdn	*SD*	Min	Max	*W*	*p*	Skewness	Kurtosis
**Preschool (predictor) variables**										
Age (months)	67.3		67.0	3.6	56	74	0.965	0.007	-0.22	-0.44
Receptive vocabulary	23.7	79.0	25.0	4.6	10	30	0.902	<0.001	-1.11	0.85
Expressive vocabulary	25.2	90.0	26.5	8.6	3	44	0.986	0.340	-0.33	-0.28
Phonological awareness	26.5	50.0	24.0	10.3	2	51	0.976	0.053	0.34	-0.18
Morphological awareness	20.1	57.4	21.0	7.9	5	34	0.946	<0.001	-0.28	-1.13
**Grade 1 (outcome) variables**										
Word accuracy	38.8	68.1	43.0	13.4	5	57	0.890	<0.001	-0.95	-0.15
Pseudoword accuracy	28.1	70.3	30.0	8.3	5	40	0.910	<0.001	-1.04	0.70
Reading fluency	41.7		39.0	16.3	9	93	0.958	0.002	0.77	1.08
Reading comprehension	14.4	68.5	15.0	3.8	2	21	0.954	0.001	-0.73	0.53
Spelling	15.2	25.3	15.0	5.2	4	29	0.973	0.029	0.44	0.50

*M% = mean percent correct; mdn = median; Min and Max refer to the lowest and highest observed value, respectively, after winsorization of outliers (see text). Shapiro–Wilks test of normality. For all measures, number of participants *N* = 104.*

**Table 2:** Intercorrelations among all variables.

	Variable		2	3	4	5	6	7	8	9	10
**Preschool (predictor) variables**											
(1)	Age (months)		0.272	0.179	0.229	0.033	0.031	0.057	0.082	0.148	0.155
(2)	Receptive vocabulary			0.420	0.270	0.307	0.160	0.211	0.230	0.440	0.206
(3)	Expressive vocabulary				0.251	0.229	0.042	0.103	0.049	0.318	0.039
(4)	Phonological awareness					0.472	0.316	0.363	0.409	0.298	0.431
(5)	Morphological awareness						0.482	0.473	0.284	0.470	0.392
**Grade 1 (outcome) variables**											
(6)	Word accuracy							0.773	0.525	0.392	0.452
(7)	Pseudoword accuracy								0.459	0.376	0.423
(8)	Reading fluency									0.332	0.693
(9)	Reading comprehension										0.360
(10)	Spelling										

*Pearson’s *r* correlation coefficients; *N* = 104; correlations of 0.193 or greater are significant at *p* < 0.05; and greater than 0.273 at *p* < 0.005.*

For each outcome variable, a hierarchical regression analysis was conducted in three steps: receptive and expressive vocabulary were entered at the first step, as proxies for language development and verbal ability in general; phonological awareness was entered at the second step, and morphological awareness at the third and final step. This was done in order to quantify the specific contribution of metalinguistic skills beyond general language skills, and in particular the specific contribution of morphological awareness beyond the – already well known – effect of phonological awareness, which in this way also acts as a proxy for general metalinguistic skill. **Table 3** displays the results of these analyses, including the total and additional variance accounted for at each step (rightmost columns), the coefficients in the final multiple regression models for each outcome variable including all predictors (leftmost columns), and the proportions of shared and unique variance accounted for by each predictor in the final models (commonality analysis; middle columns).

**Table 3:** Results of regression analyses for the longitudinal prediction of Grade 1 reading skills.

	Multiple regression			Commonality (variance)				Hierarchical regression			
Preschool predictor	β	*p*		Unique	Common	Total		Step	*R*^2^	Δ*R*^2^	*p*
**Word accuracy**											
Receptive vocabulary	0.018	0.951		<0.001	0.017	0.017		1	0.018		0.410
Expressive vocabulary	-0.148	0.336		0.007	-0.005	0.002					
Phonological awareness	0.169	0.198		0.013	0.087	0.100		2	0.106	0.089	0.002
Morphological awareness	0.748	<0.001		0.144	0.088	0.232		3	0.250	0.144	<0.001
**Pseudoword accuracy**											
Receptive vocabulary	0.047	0.794		0.001	0.029	0.030		1	0.031		0.207
Expressive vocabulary	-0.042	0.659		0.002	0.009	0.011					
Phonological awareness	0.147	0.071		0.025	0.107	0.132		2	0.138	0.108	0.001
Morphological awareness	0.409	<0.001		0.112	0.112	0.224		3	0.251	0.112	<0.001
**Fluency**											
Receptive vocabulary	0.663	0.065		0.027	0.055	0.081		1	0.081		0.014
Expressive vocabulary	-0.174	0.354		0.007	0.005	0.012					
Phonological awareness	0.527	0.001		0.084	0.108	0.192		2	0.227	0.146	<0.001
Morphological awareness	0.333	0.115		0.019	0.106	0.125		3	0.247	0.019	0.115
**Comprehension**											
Receptive vocabulary	0.215	0.007		0.052	0.127	0.179		1	0.202		<0.001
Expressive vocabulary	0.052	0.208		0.011	0.090	0.101					
Phonological awareness	0.012	0.731		0.001	0.088	0.089		2	0.231	0.029	0.056
Morphological awareness	0.168	<0.001		0.091	0.130	0.221		3	0.322	0.091	<0.001
**Spelling**											
Receptive vocabulary	0.170	0.115		0.017	0.057	0.074		1	0.075		0.019
Expressive vocabulary	-0.079	0.163		0.014	-0.007	0.007					
Phonological awareness	0.143	0.003		0.061	0.144	0.206		2	0.240	0.165	<0.001
Morphological awareness	0.223	0.001		0.085	0.153	0.238		3	0.325	0.085	0.001

**FIGURE 1:**
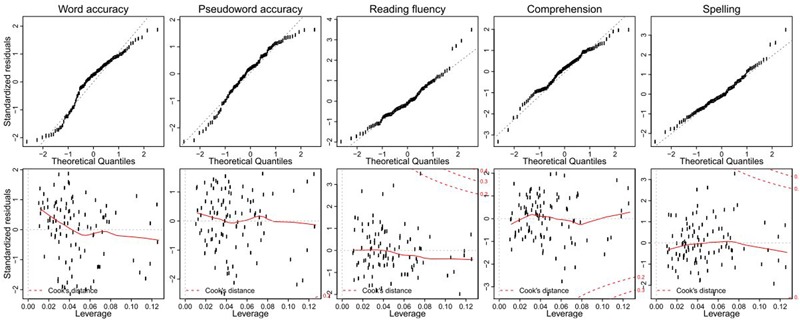
Multiple regression diagnostics. **(Top)** Quantile–quantile plots of standardized residuals for the longitudinal prediction of each outcome variable. **(Bottom)** Corresponding leverage-residual plots with overlaid smooth trend, also displaying Cook’s distance curves at values of 0.2 and 0.3.

Residual diagnostics are shown in **Figure 1**, indicating no severe deviations from normality and no overly influential data points. There was a significant unique contribution of morphological awareness, beyond vocabulary and phonological awareness, to every outcome variable except fluency, for which only phonological awareness made a significant unique contribution. The unique contribution of morphological awareness was sizeable (9–14% of variance, depending on outcome measure) and was accompanied by additional, comparable proportions of variance (9–15%) shared with the other measures, bringing up the total longitudinal predicted variance from morphological awareness to more than 20% of reading (and spelling) outcomes (except fluency).

## Discussion

In this longitudinal study we have investigated the prediction of reading and spelling outcomes near the end of Grade 1 by language and metalinguistic skills assessed in preschool 14 months earlier. Morphological awareness had a significant unique contribution to all outcome variables except reading fluency. This finding confirms the important role of morphological awareness for reading development and extends it to a younger age than usually studied.

Our results are consistent with the findings of Casalis and Louis-Alexandre (2000), who studied early reading performance longitudinally predicted by preschool phonological and morphological awareness in French, and found both a strong correlation between phonological and morphological awareness at these ages as well as longitudinal relationships between both of them and early reading. Our results are also compatible with those of Grigorakis and Manolitsis (2016), who examined the prediction of Grade 1 inflectional spelling by preschool phonological and morphological awareness in Greek, and found a significant longitudinal contribution of morphological awareness beyond phonological awareness and other control variables.

In particular with respect to spelling, one might expect an especially important role of morphological awareness in Greek (Grigorakis and Manolitsis, 2016), because, as noted above, many inflectional and derivational affixes are associated with specific spellings (and, indeed, some of them are homophonous and can only be disambiguated by spelling). This hypothesis cannot be evaluated in the current study because our strong result (Δ*R*^2^ = 0.085, *p* < 0.001) emerged using a standardized spelling test including many words with difficult stems and not giving particular weight to grammatical (i.e., inflectional suffix) spelling. This might be taken to imply that the relationship between morphological awareness and spelling is not specific to suffixes. However, our results do not speak to the issue of a suffix-specific relationship: It may well be the case that morphological awareness is especially necessary – or beneficial – for spelling inflectional suffixes, and this could only be discerned in comparison with appropriately designed spelling tests assessing performance on particular kinds of suffixes. Such studies should be performed with older children, because suffix-specific spelling knowledge is taught after Grade 1. At any rate, our findings suggest that there is also a more general sense in which early metalinguistic awareness supports the development of spelling skill. Whether this relates to language or cognitive skills required for metalinguistic task performance is not known. Future studies must use appropriate latent constructs to examine whether these observed longitudinal relationships are direct or mediated by other, more general, constructs.

Our findings seem to be somewhat at odds with those of Manolitsis (2006), who found that preschool morphological awareness longitudinally predicted Grade 1 single word reading speed, but not accuracy, after controlling for phonological awareness. We have not measured single word reading speed, so this finding is not directly comparable to our measure of text reading fluency. The difference in the longitudinal prediction of word reading accuracy is difficult to explain conclusively without more information; it may be attributable to differences in the task content or task reliabilities. In particular, two of the morphological awareness tests used by Manolitsis had internal reliabilities less than 0.70, whereas the third one was a compound inversion task, unlike the ones we used here. Despite these differences, Manolitsis also found largely shared longitudinal contributions from preschool phonological and morphological awareness to Grade 1 word reading. In other words his general pattern of findings was not inconsistent with ours.

Vocabulary made a significant unique contribution only in the prediction of reading comprehension, and this was largely accounted for by the receptive (picture selection) rather than the expressive (verbal definitions) measure. This finding is consistent with the role of vocabulary in the development of reading comprehension that has been revealed in middle elementary grades in Greek (Protopapas et al., 2007, 2013b). Vocabulary was not related to Grade 1 reading accuracy performance, even when entered in the first step of the regression. In contrast, its significant Step 1 contribution to fluency and spelling was eventually trumped by morphological awareness due to shared variance related to these outcomes. This suggests that these morphological awareness tests capture language skills variance that is relevant for reading development at this age (cf. Hjetland et al., submitted).

It has long been known that phonological and morphological awareness share much of their variance at this age (e.g., Carlisle and Nomanbhoy, 1993) and thus it is no surprise that their contribution to reading performance is largely shared (e.g., Manolitsis, 2006). In our study, phonological awareness made a significant contribution to all reading outcomes (marginal for comprehension) when entered after vocabulary, as expected. However, this was only significant for fluency and spelling, in which it included a substantial unique contribution (6–8%). In contrast, the contribution of phonological awareness to word and pseudoword accuracy and reading comprehension was largely shared with morphological awareness, ending up non-significant in the final multiple regression models. In particular, the unique contribution of phonological awareness to word and pseudoword reading accuracy, in the presence of morphological awareness, was less than 3% of the variance. One way to interpret this, going beyond any shared content between materials in phonological and morphological awareness tasks, is to consider the extent to which these morphological awareness tests may also capture more general metalinguistic skill variance that is relevant for learning to read.

This finding raises the interesting possibility that the predictive power of phonological awareness for reading development may not be entirely due to its phonological nature but perhaps in part because it concerns metalinguistic skill, which, in turn, depends on earlier language skill development. It will be necessary to examine whether this finding holds up in follow-up research, in Greek and other languages, and in a wider range of ages. One reason it has not been found in the few studies that have examined the longitudinal prediction of early reading outcomes by preschool skills may have to do with psychometric issues. Specifically, tests of morphological awareness tend to be of lower reliability than tests of phonological awareness, and therefore may not pick up all the variance that can properly be attributed to a well-defined morphological awareness construct due to measurement noise. Our study stands out for the very high reliability of both the phonological and morphological awareness measures, allowing the regression models to capture substantial proportions of the reliable variance in the dependent variables. Ideally, future studies should include multiple highly reliable tasks as indices of corresponding latent constructs in order to examine the relative contribution of different metalinguistic skills to early reading outcomes as free from measurement noise as possible.

In this work we have treated phonological and morphological awareness as unitary constructs, by combining responses from multiple subtasks examining specific aspects of these domains. This methodological choice is supported by the very high reliability of the aggregated tasks. It is also supported by strong evidence in favor of phonological awareness being a unidimensional construct (e.g., Schatschneider et al., 1999; Anthony and Lonigan, 2004; also in Greek: Papadopoulos et al., 2009, 2012). Similarly, with respect to the morphological tasks, covering both inflectional and derivational morphology, and both judgment and production tasks, Diamanti et al. (in press) found that a single factor sufficed and accounted for 59% of the total variance, consistent with a unidimensional construct for morphological awareness as well [Muse (2005) as cited in Tighe and Schatschneider (2015)].

Our study joins the long list of studies, mentioned in the introduction, in suggesting that an explicit understanding of linguistic structure is substantially predictive of future reading performance. It provides an important confirmation of the importance of morphological awareness for reading development, by testing preliterate children, for whom a reverse effect (of reading experience on the development of morphological awareness) is unlikely, and by employing highly reliable tests covering different aspects of the target construct, such as a variety of suffixes and functions and tasks of different formats and demands. In addition, our findings bring out differences in the relevance of phonological and morphological awareness for the prediction of different reading (and spelling) tasks, at least for the age tested, that is, beginner readers.

Finally, the present study raises the intriguing possibility that the general cognitive demands of metalinguistic tasks may be of utmost importance for the prediction of reading development, whereas the linguistic content of the tasks may be of secondary importance or critical for specific associations with particular reading skills. Given the increasing prominence of morphological awareness study in the reading literature, we expect that this issue will be further investigated and clarified in future comprehensive studies.

## Ethics Statement

This study was carried out in accordance with the guidelines of the American Psychological Association for treatment of human participants, with written informed consent obtained from the parents (or legal guardians) of all children participants, approval by the school authorities, and oral assent of the children prior to test administration. The protocol was approved by the Greek Ministry of Education, following positive recommendation of the Institute of Educational Policy.

## Author Contributions

VD conceptualized this study. VD, AM, AR, FA, and SP contributed to the design and implementation of data collection. AP conducted the statistical analysis of the data. VD and AP drafted the manuscript. All authors have contributed to the writing and revising of the manuscript and agree to be accountable for the content of the work.

## Conflict of Interest Statement

The preschool measures reported here form part of a commercially available screening battery (Logometro, produced by Inte^∗^Learn Multimedia Educational Applications) designed by the authors, who receive part of the proceeds from its use.
